# Clinical Pathological and Immunohistochemical Correlations in Gastric Cancer

**DOI:** 10.3390/diagnostics14131367

**Published:** 2024-06-27

**Authors:** Mihaela Andronic, Dragoș-Viorel Scripcariu, Mădălina Maria Palaghia, Ana-Maria Trofin, Valentin Bejan, Viorel Scripcariu

**Affiliations:** 1 First Surgical Clinic, Saint Spiridon Clinical Hospital, 700111 Iași, Romania; 2 Surgery Department, Regional Institute of Oncology Iași, 700483 Iasi, Romaniaviorel.scripcariu@umfiasi.ro (V.S.); 3 Faculty of Medicine, University of Medicine and Farmacy “Grigore. T. Popa” Iasi, 700115 Iasi, Romania; ana-maria.trofin@umfiasi.ro; 4 Second Surgical Clinic, Saint Spiridon Clinical Hospital, 700111 Iași, Romania

**Keywords:** gastric cancer, Her2, Ki67

## Abstract

Due to its high aggressiveness and polyclonal tumor state, stomach cancer is considered a severe health problem. In this study, we analyzed Her2 and Ki67 in correlation with patient data for the possibility of prognostic factors. The study included 48 cases of gastric tumors that had been surgically treated in a period of five years. The percentage was statistically significant for intestinal-type adenocarcinomas located in the medio-gastric region (*p* = 0.05); in the diffuse subtype, there were no Her2 positive samples, and in the mixed subtype only one out of three samples was Her2 positive. Our results confirm the existing data, and we can conclude that this link can be considered a prognostic factor in the progression and treatment effectiveness.

## 1. Introduction

Gastric cancer remains the third leading cause of cancer-related deaths worldwide, despite recent diagnosis and treatment advances [1]. There is important heterogeneity in the available literature data, due to differences in regional incidence, the diagnostic tools available, and screening methods employed. Without screening methods, diagnosis is carried out in later stages, since early stages are usually asymptomatic. This explains why, for example, in Europe, gastric cancer is diagnosed in the advanced stages of the disease; therefore, also, the 5-year survival rate in European countries is 20%, in comparison to some Asian countries, which have up to 60% 5-year survival rates. Considering the latest epidemiological data, eastern Europe has one of the highest incidences of gastric cancer [2]. Digestive cancers, taken together, are the predominated cancer types regarding incidence and mortality rates, worldwide [3]. In Romania, patients with gastric cancer are usually diagnosed in either locally advanced or metastatic stages, which nullify the possibility of radical surgery. Using a specific immunohistochemical marker to further classify each tumor subtype will improve therapeutical management with the aid of targeted immunotherapies. The present paper addresses the expression of two biomarkers—Her2 and Ki67—within a clinical and pathological framework, in order to better characterize gastric cancer in the target patients. The choice of the two biomarkers is not a novelty, with their importance being certain, although the exact role and distribution remains yet to be fully described, despite the plethora of studies available.

Her2 represents one of the first biomarkers with a clear impact in the treatment, especially in metastatic and unresectable gastric cancer, because of the use of targeted immunotherapy with trastuzumab. Although a routinely used and studied biomarker, especially with the emergence of trastuzumab-resistant tumors, its prognostic value and distribution through different phenotypes remains unclear [4,5] A couple of studies reported Her2 as a prognostic factor only in stage I gastric cancer [6]. Regarding Ki67, there is again a discrepancy between studies, with the majority of them considering this biomarker as unsuitable for prognosis. Despite this, it remains a routine biomarker, with newer data associating it with intratumoral heterogeneity [7]. Additional reliable prognostic markers, such as complex immunochemistry or DNA/RNA tests, are useful but not established as a routine diagnosis method and not widely available [8]. Therefore, in view of the important heterogeneity of the available data, the aim of the present paper is to describe the clinicopathological correlations in a study group, in order to complement the available literature data. 

## 2. Materials and Methods

We conducted a retrospective study between 2018–2022 using the patient data of 48 patients who were diagnosed with gastric cancer in General Hospital Saint Spiridon Iassy, which had Her2 and Ki67 testing. The patient database included clinical and pathological characteristics, such as age, sex, histological subtype, tumor stage, and location. All patients had confirmed preoperative pathology reports of gastric cancer, diagnosed through upper gastrointestinal biopsies, and informed consent has been obtained from all included in this study. The inclusion criteria were new cases of gastric cancer confirmed by postsurgical pathological reports of resected specimens, which was consistent with the preoperative biopsy report. All patients included in the study underwent open radical or partial D2 gastrectomy and all cancer types were adenocarcinomas. The exclusion criteria involved the inoperable cases or those who refused surgery. The study was purely retrospective and in compliance with the relevant national regulations and institutional policies, and has been approved by Medical Ethics Committee of General Hospital Sf. Spiridon Iași, Romania (No 78/31.08.2022) and by Medical Ethics Committee of The University of Medicine and Pharmacy Grigore T. Popa Iasi, Romania (No 398/12.02.2024).

All samples had hematoxylin and eosin (H&E) staining and were examined, and histopathological features were evaluated as follows:

- Gastric carcinomas were classified according to Lauren classification (intestinal and diffuse types)
- Histological grading of gastric adenocarcinomas was divided into well, moderately and poorly differentiated.
- The depth of tumor invasion, lymph node metastasis, distant metastasis and the staging of tumor were assigned using TNM classification (American Joint Committee on Cancer 7th staging system 2017).
- The lymphovascular invasion, perineural invasion, and involvement of surgical margin by the tumor were assessed.

The immunohistochemical staining was performed using Her2 and Ki67 markers in all gastrectomy specimens. We used Hofmann’s criteria for the immunohistochemical expression of Her2. Hoffmann’s criteria is based on the percentage of cells with membrane-like staining. Grades are classified as follows: grade 0: negative or <10% reactivity in tumor cells; grade 1+: weak reactivity >10% of the tumor cells; with reactivity only in part of the membrane; grade 2+: moderate reactivity in >10% of the cells, with staining across the lateral and basolateral membrane: grade 3+: strong reactivity, with intense staining of the lateral and basolateral membrane in >10% of the cells.

According to the recommendations of the International Ki67 in the Breast Cancer Working Group, positive Ki67 staining was only defined as positive nuclear staining, regardless of the staining intensity, and the average score is recorded by the assessment of complete sections including hot spots (if present). The nuclear expression of Ki67 was counted within 100 tumor cells in a hot spot area, and was graded as gastric carcinoma with low Ki67 score (≤20%) and gastric carcinoma with high Ki67 score (>20%).

## 3. Results

For the present study, from 48 analyzed patients, 34 were male (70%) and 14 were female (30%). The mean age for all the patients was 67 years. More than half (52%) of the tumors were in the medium stomach (body/fundus), 18% were proximal (cardia), and 32% were distal (antrum), and from all surgical specimens examined, 77% were well/moderately differentiated. Related to lymphatic invasion, this study identified lymph node metastasis in 75% of patients, with an average of 23 lymph nodes harvested per patient and a node ratio of 0.3. Her2 and Ki67 expressions (positive and negative) were evaluated in all 48 samples and primary gastric tumors and corresponding lymph nodes. The correlation between these two markers, demographic and clinical features are presented in Table 1, Table 2, Table 3, Table 4 and Table 5.

A total of 15 patients (31.25%) presented positive Her2 status. In our study group, there were no 3+ samples, 5 Her2 2+ (10.41%), and 10 Her2 1+ samples (20.83%). Considering the localization of the positive samples, there is no statistical correlation between the three anatomical sites.

Regarding Ki67 positive samples, they accounted for 83.33% of the study group. The percentage was statistically significant for intestinal-type adenocarcinomas located in the medio-gastric region (*p* = 0.05); for the other regions, while still an increased number, it is without any statistical significance in correlation with the Her2 positive samples. Although there appears a trend between the intestinal-type adenocarcinomas and medio-gastric region, the statistically significant association appears to be in correlation to histological type (*p* = 0.02).

A few trends can be observed in our study group, such as Ki67 positive status and advanced stages of intestinal-type carcinoma with node and vascular invasion, and Her2 is found more often in advanced stages with node invasion.

Between the two biomarkers, there is a statistical correlation, independent of the clinical and morphological characteristics (*p* = 0.03), and also there is a relatively similar distribution when considering the important morphological elements.

As seen in Table 5, there is no statistical correlation between Her2 or Ki67 status and the age of the patients in the study group, confirmed by the available data from both European and Asian studies. The percentage of positive Ki67 and Her2 status is more frequent, but without statistical correlation with the depth of tumor invasion (T3/4 stages). In these cases, Her2 is found in 30.76% of cases (*p* = 0.26), while the percentage is higher for Ki67 at 77.5% (*p* = 0.153).

The results of our study can be explained by the tumoral proliferation rate, which is in direct connection with the degree of tumor progression.

An important element revealed by our study is the strong statistical correlation between Her2 and two independent prognostic factors: perineural invasion and lymph node involvement (*p* < 0.01). Our results confirm the existing data and we can conclude that this link can be considered a prognostic factor in the progression and treatment effectiveness.

Although the current study finds no correlation between Ki67 and perineural invasion or lymph node metastasis, the only statistical correlation found for Ki67 was with tumor grading and intestinal subtype (Lauren classification).

## 4. Discussions

The traditional histological classification is based on Lauren’s criteria, which is used to categorize gastric cancer into intestinal (approximately 50%), diffuse (30%), and indeterminate (20%) types [9]. Each of these histological types have, in turn, different incidence patterns and prognoses [10]. Our present paper also acknowledges the importance of the Lauren classification; therefore, the data are correlated with each subtype.

However, this classification is inadequate in current cancer therapy framework because it does not address specific targetable molecules, such as membrane antibodies.

Human epidermal growth factor receptor 2 (Her2), also known as Neu or ErbB2, is a member of the EGFR (epithelial growth factor receptors) family and encodes the transmembrane glycoprotein p185. This tyrosine kinase, after ligand induced activation, initiates a phosphorylation signaling cascade which in turn determines proliferation, differentiation and cell growth [11]. Like many other oncogenes, in solid tumors, they are usually amplified or overexpressed [12].

There have been guidelines issued, such as Korean practice guidelines for gastric cancer, which recommend the assessment of Her2 status before the initiation of systemic chemotherapy, and, if possible, re-evaluation for recurrent or metastatic lesions [13]. Regarding the international studies carried out in Europe and Asia, respectively, where the Her2 positivity percentage is between 10–22.8% for Romania in particular, for our study we can observe the Her2 positivity value is above average, with data confirmed by the European studies.

The present study, compared to the results obtained in other areas, identifies a symmetry with already existing information, excepting a number study published in Japan [14]. Reviewing previous papers published on the expression of Her2 separately for the intestinal and diffuse subtypes, we find a prevalence in intestinal type ranging from 6.1% to 28.57%, and in the diffuse type from 0.7 to 13.43% [15,16,17]. This finding supports the evidence that the expression of Her2 has a more consistent association with Lauren’s intestinal subtype and is consistent with our findings. Regarding anatomical location, there is no statistically significant correlation in our study, comparable to similar studies conducted internationally [18,19].

When referring to the systemic therapy, trastuzumab, the first successful targeted agent against Her2, has demonstrated clinical benefits in the treatment of advanced gastric cancer. The ToGA trial (NCT01041404), a phase 3 randomized controlled trial, compared the effectiveness of a combined trastuzumab with chemotherapy (cisplatin/fluorouracil (FP) or capecitabine [XP]) versus chemotherapy alone. The results were promising, with a median overall survival (OS) of 13.8 months in the trastuzumab with chemotherapy group compared to 11.1 months in the chemotherapy alone group [20]. The results of our study can be explained by the increase in the tumor proliferation rate, which is directly proportional to the degree of tumor progression of gastric cancer and the results are compatible with international studies, such as those from Asia or America [21], but not with those from Europe, where between Her2 and tumor invasion a statistically significant value was identified [22].

Ki67 has been receiving increasing attention as an important tumor proliferation marker since 1991, when for the first time it was identified as a non-histone protein by Gerdes et al., and is highly associated with tumor development, progression, invasion, metastasis, and prognosis [23]. The data obtained from our study aligns with the existing literature data, which considers the prognostic value and clinicopathologic significance of Ki67 expression in gastric cancer patients controversial. For example, one meta-analysis published in 2017 by Luo et al. reveals that high Ki67 expression might represent an independent negative predictive biomarker for gastric cancer patients [24]. Other studies, like the one published by Xiao et al., reported that Ki67 expression is irrelevant to the prognosis of these patients [25].

Despite numerous correlations described in large studies, the expression of the antigen Ki67 alone was not significantly associated with any investigated clinicopathologic factors, and also there is no uniformity of results in the geographical areas in correlation with the degree of tumor invasion. This can be explained by the fact that the rate of tumor proliferation in gastric cancer at a certain moment does not accurately represent the type of future development of the tumor, and an inverse relationship may develop between tumor proliferation and invasion in gastric carcinoma [7].

Our study does not confirm Ki67 as an independent prognostic factor, with positive samples not being correlated with the biological behavior of stomach adenocarcinoma. These results are confirmed by the study of E Shafigh et al. [26], despite a high percentage of cases which are Ki67 positive.

Our study group also showed no significant statistical correlation between Ki67 and the sex or the location of the gastric tumor, despite the fact that a large number of positive cases are located in the body of stomach. These results are inconsistent with the results of Ko et al. [27], whose results indicated high Ki67 expression as a negative prognostic factor in patients who developed tumors located distally. Also, Naema et al. [28] revealed a significant statistical association between high Ki67 expression and gastric adenocarcinoma location in the fundus and body of the stomach.

Regarding tumor subtype, a positive expression of Ki67 was statistically significantly present in adenocarcinoma of the intestinal type (*p* = 0.05), in particular located in the middle part of the stomach, with a high rate of expression in the other locations also, but without statistical significance. The results obtained are similar to those of the study carried out by Ko et al., who found significant statistics for Ki67 positive, with the intestinal histological type of gastric carcinoma according to the Lauren classification.

The present study shows no significant statistical correlation between Ki67 level and perineural invasion, pathological grade, and local tumor invasion, with data confirmed by studies such as Xiao et al. [25], Pyo and Kim [29]. Other studies, like Badry et al. [30] and Naema et al. [28], reported a highly significant association between high Ki67 and tumor invasion (T), especially with early stages of invasion. Our study showed no statistically significant association between Ki67 expression and lymph node invasion, although the positive expression of Ki67 is very high, with the percentage reaching 83,3%. These results concur with the ones from Pyo and Kim [29]. A statistically significant relation was found in the present study between high Ki67 expression and the grade of gastric carcinoma (*p* < 0.05). These findings are in accordance with El-Gendi et al. [21], who also found the same results. For gastric cancer, there is a non-uniformity at the level of the studies carried out, and some have demonstrated that there is an association between high values of Ki67 and an unfavorable long-term prognosis [31,32], while other studies do not consider it important as a prognostic factor [19,33].

## 5. Conclusions

Gastric cancer is one of the most prevalent and most lethal malignancies, but the current TNM staging system together with clinicopathologic characteristics are not sufficient to identify cases that have a poor prognosis.

Even if this present work had a relatively limited sample size, our results reveal positive but limited clinicopathologic and prognostic values, correlated both with Her2 expression and Ki67. New research and developments, as well as studies with larger sample sizes that should adopt a prospective design and appropriate score for Her2 and Ki67 expression, may provide more information about these biomarkers for their use in early cancer detection and prognosis prediction.

Because of the important heterogeneity of the literature data, and thus the lack of consensus, our results are confirmed by the existing data, representing another piece of the diagnostic puzzle of gastric cancer.

## Figures and Tables

**Table 1:** Clinicopathological description of the study group.

Clinicopathological Characters		*N*	%
age of patient (y)	≥60	38	79.2
	≤60	10	20.8
gender	Male	35	73.0
	Female	13	27.0
anemia	Hemoglobin ≥ 11 g/dL	17	35.5
	Hemoglobin ≤ 11 g/dL	31	64.5
intervention	Surgery	48	100
	Endoscopy	0	0
location	Cardia	8	16.0
	Fundus and Body	25	52.0
	Pylorus	15	32.0
perineural invasion	Negative	13	27.0
	Positive	35	73.0
margins	Negative	36	75.0
	Positive	12	25.0
grade	Grade I	11	23.0
	Grade II	16	33.4
	Grade III	21	43.6
metastasis	Negative	43	89.5
	Positive	5	10.5
lymphovascular invasion	Negative	12	25.0
	Positive	36	75.0
pathological t	T1	6	12.5
	T2	3	6.2
	T3	19	39.6
	T4	20	42.7
pathological n (preoperative)	Negative	11	23.0
	Positive	37	77.0
stage	I	7	14.6
	II	20	41.7
	III	16	33.3
	IV	5	10.4
postoperative complications	Yes	10	20.9
	No	38	79.1
operative mortality	0	0	0

**Table 2:** Her2 and Ki67 distribution of sexes.

Distribution of Sexes							
Women				Men			
14				34			
Her2		Ki67		Her2		Ki67	
+	−	+	−	+	−	+	−
5	9	13	1	10	24	27	7

**Table 3:** Her2 Distribution for the localization of the gastric tumor in the study group.

Her2 Positive				Her2 Negative			Total
	Diffuse	Intestinal	Mixed	Diffuse	Intestinal	Mixed	
Proximal	0	1	0	4	3	0	8
Middle	0	9	0	5	9	2	25
Distal	0	4	1	5	5	0	15
Total	0	14	1	14	17	2	48

**Table 4:** Ki67 status distribution for the localization of the gastric tumor in the study group.

Ki67 Positive				Ki67 Negative			Total
	Diffuse	Intestinal	Mixed	Diffuse	Intestinal	Mixed	
Proximal	4	3	0	0	1	0	8
Middle	4	17	1	1	1	1	25
Distal	5	6	0	0	3	1	15
Total	13	26	1	1	5	2	48

**Table 5:** Relation between Her2 and Ki67 expression and clinicopathological characters.

Characteristics	*N* = 48	KI 67		*p*-Value	HER2		*p*-Value
		Positive	Negative		Positive	Negative	
Gender				*p* > 0.05			*p* > 0.05
Male	34	27	7		10	24	
Female	14	13	1		5	9	
Age (year)				*p* > 0.05			*p* > 0.05
≤60	9	7	2		4	5	
≥60	39	33	6		11	28	
Tumor Pathological				*p* > 0.05			*p* > 0.05
T1/T2	9	8	1		3	6	
T3/T4	39	32	7		12	27	
Regional lymph node metastasis				*p* > 0.05			*p* < 0.05
Positive	36	28	8		13	23	
Negative	12	12	0		2	10	
Pathology grading				*p* < 0.05			*p* > 0.05
High differentiation	21	17	4		7	14	
Middle differentiation	16	14	2		6	10	
Low differentiation	11	9	2		2	9	
Perineural invasion				*p* > 0.05			*p* < 0.05
Positive	35	12	1		2	11	
Negative	28	7	7		13	22	

## Data Availability

Data can be made available through e-mailing the corresponding author.
